# A Research Agenda for Helminth Diseases of Humans: Towards Control and Elimination

**DOI:** 10.1371/journal.pntd.0001547

**Published:** 2012-04-24

**Authors:** Boakye A. Boatin, María-Gloria Basáñez, Roger K. Prichard, Kwablah Awadzi, Rashida M. Barakat, Héctor H. García, Andrea Gazzinelli, Warwick N. Grant, James S. McCarthy, Eliézer K. N'Goran, Mike Y. Osei-Atweneboana, Banchob Sripa, Guo-Jing Yang, Sara Lustigman

**Affiliations:** 1 Lymphatic Filariasis Support Centre, Department of Parasitology, Noguchi Memorial Institute for Medical Research, University of Ghana, Legon, Ghana; 2 Institute of Parasitology, McGill University, Montreal, Canada; 3 Department of Infectious Disease Epidemiology, School of Public Health, Faculty of Medicine, Imperial College London, London, United Kingdom; 4 Onchocerciasis Chemotherapy Research Centre, Hohoe Hospital, Hohoe, Ghana; 5 High Institute of Public Health, Alexandria University, Alexandria, Egypt; 6 Department of Microbiology, Universidad Peruana Cayetano Heredia, Lima, Peru; 7 Department of Maternal and Child Nursing and Public Health, School of Nursing, Federal University of Minas Gerais, Belo Horizonte, Minas Gerais, Brazil; 8 The Nematode Functional Genomics Laboratory, La Trobe University, Victoria, Australia; 9 Queensland Institute of Medical Research, University of Queensland, Herston, Australia; 10 Laboratoire de Zoologie et de Biologie Animale, Université de Cocody, UFR Biosciences, Abidjan, Côte d'Ivoire; 11 Council for Scientific and Industrial Research (CSIR), Department of Environmental Biology and Health, Water Research Institute, Accra, Ghana; 12 Tropical Disease Research Laboratory, Division of Experimental Pathology, Department of Pathology, Khon Kaen University, Khon Kaen, Thailand; 13 Department of Schistosomiasis Control, Jiangsu Institute of Parasitic Diseases, Meiyuan Yangxiang, Wuxi, People's Republic of China; 14 Laboratory of Molecular Parasitology, Lindsley F. Kimball Research Institute, New York Blood Center, New York, New York, United States of America; University of Oklahoma Health Sciences Center, United States of America

## Abstract

Human helminthiases are of considerable public health importance in sub-Saharan Africa, Asia, and Latin America. The acknowledgement of the disease burden due to helminth infections, the availability of donated or affordable drugs that are mostly safe and moderately efficacious, and the implementation of viable mass drug administration (MDA) interventions have prompted the establishment of various large-scale control and elimination programmes. These programmes have benefited from improved epidemiological mapping of the infections, better understanding of the scope and limitations of currently available diagnostics and of the relationship between infection and morbidity, feasibility of community-directed or school-based interventions, and advances in the design of monitoring and evaluation (M&E) protocols. Considerable success has been achieved in reducing morbidity or suppressing transmission in a number of settings, whilst challenges remain in many others. Some of the obstacles include the lack of diagnostic tools appropriate to the changing requirements of ongoing interventions and elimination settings; the reliance on a handful of drugs about which not enough is known regarding modes of action, modes of resistance, and optimal dosage singly or in combination; the difficulties in sustaining adequate coverage and compliance in prolonged and/or integrated programmes; an incomplete understanding of the social, behavioural, and environmental determinants of infection; and last, but not least, very little investment in research and development (R&D). The Disease Reference Group on Helminth Infections (DRG4), established in 2009 by the Special Programme for Research and Training in Tropical Diseases (TDR), was given the mandate to undertake a comprehensive review of recent advances in helminthiases research, identify research gaps, and rank priorities for an R&D agenda for the control and elimination of these infections. This review presents the processes undertaken to identify and rank ten top research priorities; discusses the implications of realising these priorities in terms of their potential for improving global health and achieving the Millennium Development Goals (MDGs); outlines salient research funding needs; and introduces the series of reviews that follow in this *PLoS Neglected Tropical Diseases* collection, “A Research Agenda for Helminth Diseases of Humans.”

## Introduction

Human helminthiases affect mostly populations in sub-Saharan Africa, Asia, and the Americas [1]. The affected populations are typically largely marginalised, live in low-income settings, and account for over 1 billion people infected with one or more helminth species. These infections are associated with low work productivity, poor cognitive performance, and slow socioeconomic development, thereby contributing to accentuate poverty and inequality [1], [2]. Despite their insidious effects at the individual and societal levels, helminth infections and their associated disease sequelae have not received as much research and investment attention as other acute or life-threatening conditions, a fact that has placed human helminthiases in the category of neglected tropical diseases (NTDs). However, the collective morbidity that these diseases cause is considerable and comparable to that caused by malaria, HIV/AIDS, or tuberculosis [2], and they are also responsible for excess mortality [3]–[5].

Consequently, over the past three decades (straddling the last quarter of the 20th century and the first decade of this century), increased acknowledgement of the impact of their burden on human communities has led to an expansion of the number of large-scale control and elimination programmes against helminth infections, aiming to eliminate the public health problem they pose (morbidity reduction), or the infection reservoir (parasite elimination). The relatively limited arsenal of available tools for intervention against helminthiases, the extent and heterogeneity of their geographic distribution, and the sheer magnitude of the overall task, have meant that morbidity control rather than parasite elimination has been the initial target for most programmes. Examples of these, mostly vertical, single-disease interventions are the Onchocerciasis Control Programme in West Africa (OCP), the African Programme for Onchocerciasis Control (APOC), the Schistosomiasis Control Initiative (SCI), and the Partners for Parasite Control (PPC). Exceptions to this are those campaigns that started with a clear mandate of eradication (e.g., Guinea Worm Eradication Programme) or parasite elimination (the Joint Research Management Committee [JRMC] for Schistosomiasis Elimination in China, the Global Programme to Eliminate Lymphatic Filariasis [GPELF], and the Onchocerciasis Elimination Program for the Americas [OEPA]). However, as morbidity reduction initiatives advance, their goals have become more ambitious, aiming at elimination where deemed feasible. Multiple helminth infections, affecting the same populations parasitised by more than one species, are also common [5], though the burden of disease due to co-morbidity remains largely unknown [6]. The increased recognition of this co-endemicity and polyparasitism has prompted the establishment of integrated, multi-helminth, and multi-NTD control strategies.

Whilst this shift in focus is understandable (essentially indefinite programmes risk fatigue of sponsors and populations, and there is a need to optimise efforts and resources), the question remains as to whether the scientific and public health communities truly have the knowledge and the tools matched to the task of controlling/eliminating human helminthiases regionally, globally, and in an integrated manner. It is with the objective of contributing to answering this question that the Disease Reference Group on Helminth Infections (DRG4) was established by the Special Programme for Research and Training in Tropical Diseases (TDR). The infections within the remit of this group include the filariases (onchocerciasis and lymphatic filariasis), trematodiases (intestinal and urinary schistosomiasis; food-borne, liver fluke infections), the soil-transmitted helminthiases (intestinal nematode infections), and cestode infections (taeniasis/cysticercosis). (Note that some of these infections are zoonoses [e.g., Asian schistosomiasis, food-borne trematodiases, taeniasis/cysticercosis] and recognised under the umbrella of neglected zoonotic diseases [NZDs] by the first World Health Organization [WHO] report on NTDs [7]. The various aspects of veterinary public health as one of the pivotal strategies for the prevention and control of NZDs have been discussed by DRG6 [Table S1] and summarised in their recent review [8], and therefore are not addressed extensively by DRG4.)

Firstly, some key research findings leading to (or stemming from) the establishment of some of the above mentioned programmes are outlined (for further detail on the problem of helminthiases, large-scale control programmes past and present, and available tools for intervention against these infections, see Lustigman et al. [9] and Prichard et al. [10] in this collection of reviews). Secondly, the DRG4 is introduced, and the processes that the group used to identify research gaps and key priority research areas are described. Finally, some of the issues that challenge sustained control and elimination of helminthiases are discussed, and the rationale is presented for the review articles contained within this *PLoS Neglected Tropical Diseases* collection, “A Research Agenda for Helminth Diseases of Humans”. Box 1 lists the abbreviations used in this article.

**Box 1. List of Abbreviations**

**APOC,** African Programme for Onchocerciasis Control

**DALY,** disability-adjusted life year

**DRG4,** Disease Reference Group on Helminth Infections

**G-FINDER,** Global Funding of Innovation for Neglected Diseases

**GPELF,** Global Programme to Eliminate Lymphatic Filariasis

**JRMC,** Joint Research Management Committee for Schistosomiasis Elimination in China

**LF,** lymphatic filariasis

**MDA,** mass drug administration

**MDGs,** Millennium Development Goals

**M&E,** monitoring and evaluation

**NTDs,** neglected tropical diseases

**NZDs,** neglected zoonotic diseases

**OCP,** Onchocerciasis Control Programme in West Africa

**OEPA,** Onchocerciasis Elimination Program for the Americas

**PAHO,** Pan American Health Organization

**PPC,** Partners for Parasite Control

**SCI,** Schistosomiasis Control Initiative

**R&D,** research and development

**STHs,** soil-transmitted helminthiases

**TDR,** Special Programme for Research and Training in Tropical Diseases

**UNICEF,** United Nations Children's Fund (formerly United Nations International Children's Emergency Fund)

**UNDP,** United Nations Development Programme

**WHO,** World Health Organization

## Research for Control and Elimination of Human Helminthiases

Undoubtedly, there has been much scientific advancement in our understanding of the biology and epidemiology of helminth infections that has underpinned control efforts. For the OCP, early research on *Onchocerca–Simulium* complexes, on epidemiological and parasite heterogeneity regarding blindness patterns [11], and on cytotaxonomy, distribution, ecology, and flight range of the various members of the *Simulium damnosum* complex [12] helped to demarcate original areas under (vector) control and further extensions. Importantly, operations research has been crucial to solving some of the programmatic issues confronted by the programmes throughout their implementation, such as the development of insecticide resistance and reinvasion of areas under control by infective flies [13]. (In the context of this paper [and others in this collection], operations research is used to refer to the utilisation of relevant biological knowledge and appropriate and updated technologies by large-scale parasite control initiatives for the deployment of effective and optimal strategies aimed to reduce the parasite burden, transmission, and morbidity of poverty-related infectious diseases in general and helminthiases in particular.)

A further example is that of the licensing of ivermectin for human use in 1987, and its subsequent donation by Merck & Co. for as long as needed against river blindness, which prompted research into the “why”, “where”, and “how” to control onchocerciasis on a large scale with chemotherapy in addition to vector control or as the only measure being implemented [14]. Consequently, research on the social importance of onchocercal skin disease provided the justification for extending control to other endemic areas outside the savanna regions of West Africa, where blindness had been the major motivation for control by the OCP. This extension made it possible for millions of people to benefit from the control efforts implemented by APOC. The development of the rapid epidemiological assessment and mapping methods that ensued [15]–[17] also facilitated demarcation of areas prioritised for ivermectin distribution (e.g., meso- and hyperendemic areas), and more recently, identification of areas where specific management strategies or novel control tools are needed (e.g., areas co-endemic for onchocerciasis and loiasis with a high risk of severe adverse events if ivermectin is administered) [18].

Sensitive and specific parasite antigen tests for *Wuchereria bancrofti*, developed in a rapid card format, made the mapping of the distribution of Bancroftian filariasis operationally feasible [19]. This, together with the donation or affordability of drugs for safe mass administration in combinations highly efficacious against the microfilariae and affecting the adult worms to a lesser extent (but importantly, prolonging the period when the density of microfilariae, which can be transmitted to vectors, remains low), promoted the idea that elimination of lymphatic filariasis (LF) was attainable as a public health goal [20]. As the GPELF has advanced, and challenges have been encountered, priority areas for programmatic research have been articulated, including 1) refinement of tools and evidence-based measurements of programme success for stopping mass treatment in diverse settings and according to vector–parasite complexes; 2) increased efforts to enhance programme effectiveness by improving MDA coverage and compliance, using vector control where feasible, and integrating with other programmes where appropriate; 3) improved clinical management for individuals with LF disease; and 4) protection of the LF programme by monitoring drug resistance and developing new drugs [21], [22].

For schistosomiasis, access to clean water and adequate sanitation systems, snail (intermediate host) control, and treatment of infections in humans (and other mammalian hosts where possible) are the major intervention tools. However, water and sanitation are still lacking in many of the poorest communities, and molluscicides are difficult to use and often environmentally unacceptable. Therefore, “preventative chemotherapy”, aimed at reducing the intensity of infection and subsequent morbidity, has become the tool of choice [23]. Currently, preventative chemotherapy is recommended by the WHO against schistosomiasis and soil-transmitted helminthiases (STHs) [24]. The SCI adopted integrated schistosomiasis and intestinal worm control in their strategy to support national control programmes in sub-Saharan Africa [23]. The SCI has embedded research into this strategy from the outset, supporting the mapping of the infections in the participating countries [25]; developing appropriate protocols for monitoring and evaluation (M&E) [26]; using mathematical models for quantification of impact [27]; investigating parasite population genetic structure and the possibility of drug resistance [28], and furthering understanding of the relationship between infection and morbidity indicators [29]. For other diseases under our remit (e.g., food-borne trematodiases and taeniasis/cysticercosis), we refer the readers to [7]–[10], and the “One Health” approach that has been promoted extensively in the past couple of years as an essential way to control and eliminate zoonotic infections [30].

Despite the pivotal role that basic and operations research has obviously played towards the attainment of helminthiasis control and elimination, the current research and development (R&D) agenda for these infections, let alone the funding to pursue it, is still insufficient, another feature of the NTDs. Most programmes face major deficiencies in the availability of novel intervention and diagnostic tools, and in the fundamental knowledge of helminth biology that can serve as a platform from which to implement strategies for optimal combination of existing interventions, and to help with the development of the novel tools that are required.

## The Disease Reference Group on Helminth Infections (DRG4)

TDR has a 10-year strategy (http://www.who.int/tdr/about/strategy/en/) to foster “an effective global research effort on infectious diseases of poverty in which disease-endemic countries play a pivotal role” [31]. As part of this strategy, TDR has established a global research “think-tank” of 125 international experts grouped into ten thematic and disease-specific groups to review evidence continually and systematically, assess research needs and, following periodic national and regional stakeholder consultations, set research priorities for accelerating the control of infectious diseases of poverty. Working in ten disease-specific and thematic reference groups (DRGs/TRGs; Table S1), one of which is the DRG4, these experts are crucial contributors to TDR's stewardship mandate for the acquisition and analysis of information on infectious diseases of poverty (http://whqlibdoc.who.int/hq/2007/TDR_GEN_07.1_EN_Rev.1_eng.pdf). Their work is ultimately intended to promote control-relevant research, achieve research innovation, and enhance the capacity of disease-endemic countries to tackle public health problems related to the disproportionate burden of infectious diseases among the poor.

The DRG4 started with 14 members (the authors of this review), recognised as academic or public health leaders selected from research institutions, international bodies, public health organisations, and governmental organisations. (The group is chaired by SL, co-chaired by BAB, MYO-A is the Career Research Fellow, and the Core Writing Group comprises SL, M-GB, RKP, and BAB.) Its mandate is to a) obtain, evaluate, and synthesise scientific information on global research activities and challenges in research on helminth infections and other emerging helminth diseases of public health importance encompassing onchocerciasis, LF, STHs, schistosomiasis, food-borne trematodiases, and taeniasis/cysticercosis; b) act as a think tank for research on helminth diseases of public health importance, including advocacy; c) communicate their findings and recommendations on a regular basis via shared communities-of-practice (e.g., http://www.TropIKA.net and/or other appropriate, open-access publication forums); d) develop and implement a workplan according to the TDR General Operations Guide of Disease-Specific (DRG) and Thematic Reference Groups (TRG) for Research on Infectious Diseases of Poverty; and e) prepare annual reports for peer-review and future use on http://www.TropIKA.net and/or others, and for the Global Report for Research on Infectious Diseases of Poverty (http://www.who.int/tdr/stewardship/global_report/en/index.html).

In preparation for the first meeting of the DRG4 in Burkina Faso, in January 2010, all members of the group were asked to prepare white papers and oral presentations (Table S2) summarising scientific information on current activities in global research on helminth infections; highlighting progress; identifying knowledge gaps, needs, and challenges; and suggesting priorities for future global research based on four questions: what is known; what (existing) research has not been used or applied; what is not known; and what research is needed. Text S1 describes the meetings of the group, the stakeholder consultations that preceded these meetings, and the methodology used for identification, prioritisation, and ranking of research gaps.

**Figure 1:**
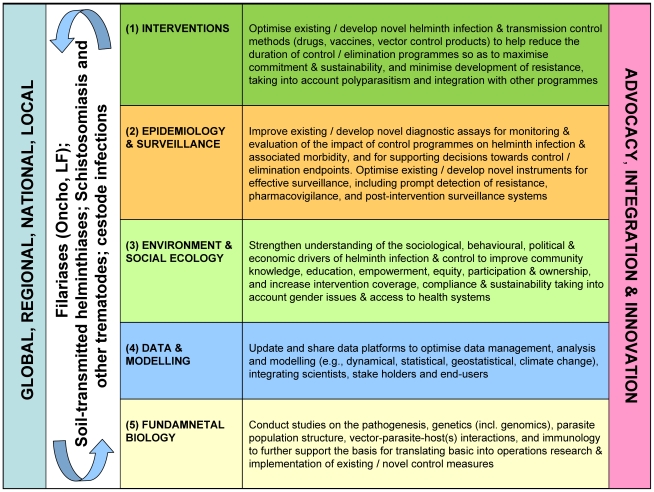
Five major core themes identified by DRG4. Umbrella priorities identified by the Disease Reference Group on Helminth Infections (DRG4) for the control and elimination of human helminthiases under its remit, namely, onchocerciasis, lymphatic filariasis, schistosomiasis, soil-transmitted helminthiases, food-borne trematodiases, and taeniasis/cysticercosis.

At the Burkina Faso meeting, five main core themes of 1) interventions, 2) epidemiology and surveillance, 3) environmental and social ecology, 4) data and modelling, and 5) basic (fundamental) biology (Figure 1) were identified as umbrella priorities to further support the control and elimination of helminthiases. Other themes within these umbrella priorities were also identified, and three areas—advocacy, integration, and innovation—cutting across all five core themes were considered (Figure 1). It was clear that within each of the core themes issues at global, regional, national, and local levels needed to be addressed. Box 2 lists the underlying values and criteria for ranking that were followed to prioritise research gaps, and Table 1 presents the ten top priority research areas identified. A league table (Table S3) was prepared for the ten priority research areas as described in Text S1. Text S2 presents the projected time horizons for the achievement of the priority research areas identified and discusses their potential impact on global health and the attainment of the Millennium Development Goals (MDGs). The outcomes are summarised in Table S4.

**Box 2. Underlying Values and Criteria for Ranking Research Areas**

***Underlying values***

- Curative or preventative relevance at patient/community level
- Public health relevance/impact on population health
- Pro-poor/poverty alleviation
- Millennium Development Goals and/or other relevant global targets
- Health security relevance
- Inter-sectoral
- Equity/gender and equity/social justice
- Positive risk-benefit ratio
- Feasibility
- Universality
- Global public good
- Innovation

***Criteria for ranking***

- Potential public health impact (by disease burden reduction)
- Size of population benefiting from research
- Feasibility (Cost benefit)
- Economic implications (Cost effectiveness)
- Equity implications
- Equality implications

**Table 1:** The Top Ten Priority Research Areas Identified by DRG4.

Core Theme^a^	Priority^b^	Description of Priority
(1)	1	Optimise existing intervention tools to maximise impact (taking into account polyparasitism) and sustainability. The tools include pharmaceuticals, vaccines, vector control, and eco-health approaches (access to clean water and sanitation, improved nutrition, education). Sustainability depends on minimising selection for drug resistance and maintaining community support for adequate coverage and compliance.
	2	Develop novel control tools that will improve impact and sustainability. The tools include new pharmaceuticals, vaccines, vector control methods, and eco-health approaches.
(2)	3	Improve existing/develop novel diagnostic tests, with particular reference to their performance regarding sensitivity, specificity, multiplex capacity, and ability to measure infection intensity/active infection. Sensitivity and specificity are mostly important to enable diagnosis of infection at low prevalence in elimination settings and to confirm cure/absence of particular infection.
	4	Standardise and validate methodologies and protocols for diagnosis in monitoring and evaluation (M&E) settings.
(1, 3)	5	Develop strategies incorporating delivery of multiple and combinations of interventions at various (individual, community, district, national) levels to maximise sustainability of control programmes in general and of integrated control programmes in particular.
(3)	6	Develop strategies (taking gender issues into account) to increase community participation, ownership, and empowerment, as well as equity in access by communities and risk groups to health services.
(4)	7	Develop and refine mathematical models to investigate relationships between infection and morbidities to aid programmes aiming to reduce the burden of disease (elimination of public health problem). Such models need to take into account cumulative effects of chronic disease for evaluation of disease burden and the impact on such burden of control interventions.
	8	Increase use and application of mathematical models to aid M&E, surveillance, elimination efforts, and the design of sampling protocols as well as the monitoring of intervention efficacy, including drug resistance. These models should be linked to economic impact studies of the diseases and cost-effectiveness analyses of the interventions, their combinations, and their alternatives.
(5)	9	Define the determinants and impact of parasite modulation of the host–parasite relationship, including impact on the host response to concurrent infection with other helminth and non-helminth pathogens and to vaccination, and parasite responses, including immune responses to interventions.
	10	Annotate parasite genomes and transcriptomes and develop tools for parasite functional genomics (and other “omics”) in key species.

a Core themes are (1) control interventions; (2) epidemiology and surveillance; (3) environmental and social ecology; (4) data and modelling; (5) basic (fundamental) biology (see Figure 1).

b Numbering of the ten top priories does not reflect order of importance; instead, they are organised according to core theme; all the (inter-connected) priorities are to be addressed in parallel as each priority will benefit from accomplishing the others.

## Challenges in the Control and Elimination of Human Helminthiasis

Although there has been much progress in the research and control of human helminthiases [32]–[38], and in addressing the operational issues and research needs that arise during the implementation of the programmes [39], major obstacles remain that challenge the global public health community and for which fundamental and applied research is urgently needed. The following outlines salient areas that will be covered in greater depth, together with others, in the reviews presented in this collection.

### Mapping

There is a need for more accurate and updated disease mapping. An example is that of LF, for which the tools available for rapid assessment of infection prevalence (immunochromatographic card tests for detection of circulating filarial antigen) and incorporation of areas into the GPELF (when prevalence surpasses 1%) have proven unreliable under field conditions [40], [41]. The advent of geographical information systems, more accurate national and regional mapping of infections, and the application of model-based geostatistics have helped various programmes to demarcate areas in need of control [42]. These maps, however, should be dynamic entities that change with time as control progresses, necessitating refinement of tools for updating the original disease maps (e.g., through linking geostatistical with transmission dynamics models).

### Diagnostics

Current diagnostics, as used in field and routine settings, provide rather inaccurate (and indirect) measures of infection prevalence and intensity that are subject to a great deal of variability and measurement error. Yet, they provide the basis for most of the epidemiological assessment upon which control programmes are based, and for assessment of drug efficacy by phenotypic means. Following several years of control and elimination programmes the problem compounds, as average infection levels will have fallen to very low levels, masking increased heterogeneity and making it difficult to distinguish true absence of infection from false negatives. There is a need to better account for variability in infection measures [43], compare and optimise (including comparative costs in operational settings) the diagnostic performance of currently available tests for epidemiological and drug efficacy monitoring [44]–[46], and to develop novel assays that respond to the changing needs of ongoing control programmes [47].

### Monitoring and Evaluation (M&E) and Surveillance

Most programmes require demonstrable evidence of impact, either as vertical programmes delivered by mobile teams, community-directed interventions, or integrated programmes with other NTD and infectious disease interventions. There is a need to design robust cohort studies that allow quantification of changes in incidence of infection [27] and disease [48]; to develop and cost sampling protocols for integrated interventions in co-endemic areas [49], ; to assess drug efficacy and develop genetic markers for investigation of parasite population structure and possible changes effected by chemotherapeutic pressure [51]–[53]; to optimise and develop diagnostic and analytical (quantitative) tools for determination of programme endpoints (for elimination of the public health burden and/or the infection reservoir) [54]; and to implement systems for surveillance [55]. Mathematical models can support M&E [27], prompt detection of anthelmintic resistance [56], and elimination efforts in a variety of settings [57].

### Coverage of and Adherence to the Interventions

Optimum intervention coverage (with MDA and/or any other intervention such as vector or snail control) is required for the success of control or elimination programmes. Intervention coverage is a key determinant for the programmes to achieve their targets [58]. However, in several countries (e.g., India), a gap between coverage and compliance has been observed in the treatment of LF [59]. The contribution of non-compliant individuals to transmission has not been quantified but it could be considerable (for instance, if those who do not adhere to treatment are heavily infected individuals who experienced unpleasant side effects on first treatment), and systematic non-compliance represents a potential threat to elimination. Research to understand the determinants of compliance in conjunction with studies on socio-behavioural, educational, and political drivers of programme acceptability and adherence [60], [61] is urgently required given the prolonged duration of intervention that is often needed. This is also important given the increasing trend for integrating control programmes that target multiple infections. Integration of interventions, at least in the initial phases of transition from well-run vertical programmes to integrated programmes, may temporarily decrease coverage [22], [61], but see [62].

### Modes of Delivery

Current MDA strategies are more suited to rural communities. It has been argued that the structures in rural, sedentary communities favour this type of approach, particularly when interventions are community directed [62]. However, a large number of helminth diseases also occur in urban areas and in pastoral and nomadic populations, as well as in conflict and post-conflict areas. The social and cultural structures in these groups of populations and areas are quite different from those in the rural situation. It will be important to undertake operations research to determine the best strategies for MDA and morbidity management in such groups.

### Intervention Tools

The donation by pharmaceutical companies of broad-spectrum anthelmintics (ivermectin, albendazole, mebendazole), the increased affordability of generic drugs produced in disease-endemic countries (diethylcarbamazine, praziquantel) in comparison with (seemingly) more expensive and cumbersome vector or intermediate host control, and the lack of sanitation infrastructure and economic development in many endemic areas, have made preventative chemotherapy the cornerstone of helminth control. However, the reliance on very few drugs (mainly developed against parasites of veterinary importance rather than for treatment of humans), the fact that most drugs fall short of being highly efficacious and in some cases (the filarial nematodes) do not kill the adult worms, and that dosages, combinations, and frequency of administration have not, by and large, been optimised, means that programmes lack the best tools for the job [63], [64]. There is an urgent need for a macrofilaricidal drug or drug combinations for onchocerciasis and LF. Antibiotic treatment regimes that deplete these parasites of their bacterial (*Wolbachia*) endosymbionts have shown promising results by leading to adult worm mortality/sterility [65], but the length of treatment that is required and the contraindications that apply at present make current regimens less suitable for community-directed intervention (but see [66]). Vector control, in combination with anthelmintic treatment, can play an important role in reducing vector density to levels below threshold biting rates and therefore aid elimination, reduce reinfection rates and programme duration, and help minimise the spread of anthelmintic resistance [67]–[69]. Integration of anthelmintic and antimalarial interventions can make use of the fact that in some areas both *Plasmodium* spp. and *W. bancrofti* are transmitted by the same *Anopheles* mosquito species, so vector control for malaria can have an impact on LF transmission [70], and distribution of anthelmintic treatment (e.g., ivermectin) in areas co-endemic for LF and/or onchocerciasis and malaria may have an impact on malaria transmission [71]. Research is needed to determine optimal combinations of anthelmintics in particular and interventions in general, and mathematical models can help in assessing these for a variety of epidemiological scenarios, endemicity levels, transmission intensities, and vector species composition, as has been done in malaria [72].

### Funding for Research and Development (R&D)

The G-FINDER (Global Funding of Innovation for Neglected Diseases) survey published in 2009 [73], reviewed by Moran et al. [74], and updated in 2010 [75], aims to report accurate and comparable R&D investment figures for infectious diseases of poverty, contrasting the “Big Three” (HIV, tuberculosis, and malaria) with the NTDs. The report noted that research funding is highly concentrated towards the former, but not necessarily correlated with disease burden. For instance, HIV, tuberculosis, and malaria accounted for 125 million disability-adjusted life years (DALYs) in low- and middle-income countries in 2004, and received nearly 80% of the total funding. By contrast, pneumonia and diarrhoeal diseases accounted for 165 million DALYs in the same countries and year, yet received less than 6% of the total funding. In particular, helminth infections received less than half the funding for diseases caused by kinetoplastid protozoans (leishmaniasis and trypanosomiasis), although their disease burden was three times as high (12 million DALYs in 2004 for the helminthiases compared to 4 million DALYs for the infections caused by these protozoans). In the G-FINDER survey, R&D products include drugs, vaccines (preventative and therapeutic), and diagnostics; vector control products (pesticides, biological control agents, and vaccines targeting animal reservoirs); and platform technologies (adjuvants, diagnostic platforms, and delivery devices). Helminth infections specifically were considered to require the following range of R&D activities: basic research and drugs for all listed infections (schistosomiasis, LF, onchocerciasis, STHs, taeniasis/cysticercosis); vaccines and novel diagnostics for schistosomiasis, onchocerciasis, strongyloidiasis, and hookworm infection; and vector/intermediate host control products for schistosomiasis, onchocerciasis, LF, and taeniasis. Although the proportion of total R&D funding allocated to helminthiases has shown a slight upwards trend (2.0% in 2007, 2.3% in 2008, 2.5% in 2009) [75], investment dwarfs in comparison to the 72% corresponding to the Big Three, positioning helminth infections in the 7th place of the R&D investment ranking table, preceded by HIV, malaria, tuberculosis, diarrhoeal diseases, dengue, and leishmaniasis/trypanosomiasis [75].

Lack of knowledge of the health and socioeconomic impact of helminth diseases, and appropriate appraisal of the cost-effectiveness of control interventions, is generally one of the biggest obstacles to obtaining funds for investment in basic and applied research [76]. Although many examples of highly cost-effective interventions to control helminth diseases exist, our understanding of the full economic effect that these diseases have on individuals, households, and nations remains incomplete [77], and is particularly scanty regarding, for instance, the food-borne trematodiases (e.g., the recent and excellent review by Conteh et al. [77] does not include data on these infections in their table on economic costs of selected NTDs). One of the obstacles to assessing the overall burden of helminth disease is its reliance on the DALY metrics, whose main determinant is the mortality rate. Instead, helminth diseases are characterised by a lifetime of disablement. Therefore, DALYs do not adequately quantify the chronic nature of these diseases [6]. As DALY estimates currently attributed to individual helminth diseases are deemed to be too low, the incremental cost-effectiveness ratio of any economic evaluation will be underestimated [77], [78]. It is therefore essential that estimates of numbers infected and disease burden are refined and updated evidence is presented on the costs, cost-effectiveness, and financing of different strategies to monitor, control, or reduce morbidity and mortality associated with these diseases [77], [79], [80]. This problem is even more pronounced for the zoonotic helminthiases, as there is a lack of reliable qualitative and quantitative data on the burden of such diseases. Importantly, these data need to go beyond the DALY to measure and incorporate economic and health burdens resulting from production losses due to disease in livestock [8], [81].

## A Research Agenda for Helminth Diseases of Humans

The five core themes identified (Figure 1) form the basis of the review articles prepared by DRG4 members for this *PLoS Neglected Tropical Diseases* collection. The reviews are intended to discuss in depth the issues pertaining to each of the umbrella priorities and expand on the research gaps and needs, presenting a detailed R&D agenda for human helminthiases for each of the core themes that is beyond the scope of this introduction to the collection.

In the first review, “The Problem of Helminthiases”, Sara Lustigman and co-authors [9] discuss how the disproportionate burden of disease caused by helminth infections in the poorest communities contributes to a vicious cycle of infection, poverty, decreased productivity, and inadequate socioeconomic development. The review provides an overview of the forces driving the persistence of helminthiases as a public health problem despite the many control initiatives that have been put in place, identifies the main obstacles that impede progress towards their control and elimination, and discusses recent advances, opportunities, and challenges for the understanding of the biology, epidemiology, and control of these infections.

In the second review, “Intervention for Control and Elimination”, Roger Prichard and co-authors [10] discuss the status of current intervention tools for the control and elimination of helminth infections that are useful but not adequate in all settings. Recent advances and remaining obstacles drive the need for an R&D agenda to ensure that the appropriate interventions (drugs, vaccines, vector control, environmental improvement) are available for use in helminth control and elimination in an optimised and timely manner, that novel anthelmintics are developed, and that resistance to drugs, insecticides, or vaccines is minimised.

In the third review, “Diagnostics for Control and Elimination Programmes”, James S. McCarthy and co-authors [82] discuss how diagnostic tools appropriate for implementation, M&E, and surveillance of interventions to control helminth infections are crucial to their success. However, the development and implementation of diagnostics has not been uniform across diseases. Pilot studies on proof of concept of new and promising diagnostic technologies have not been followed by much needed product development, so in some settings diagnosis continues to rely on insensitive and unsatisfactory parasitological or serodiagnostic techniques. In this review, current and under development diagnostic technologies for control and elimination of helminth infection are reviewed and critical gaps and opportunities are identified.

In the fourth review, “Social Ecology, Environmental Determinants, and Health Systems”, Andrea Gazzinelli and co-authors [83] focus on the environmental, social, behavioural, and political determinants of human helminth infections and outline a research and development agenda for the socioeconomic and health systems research required for the development of sustainable control programmes. Factors related to poverty, migration, and the environment (including ecological factors, climate change, water resources, and housing conditions), as well as issues related to polyparasitism, community participation, and equity in access to health services (including gender, intersectoral collaboration, and interdisciplinary research) are examined. It is concluded that research on social and environmental determinants can contribute significantly to the prevention and control of helminth diseases and thus demands greater attention by the public health community.

In the fifth review, “Modelling for Control and Elimination”, María-Gloria Basáñez and co-authors [84] argue that although mathematical modelling of helminth infections has the potential to inform policy and guide research for the control and elimination of human helminthiases, this potential has yet to be realised. A summary of the development of mathematical models for helminthiases is presented and current efforts are discussed according to the role that models can play at various stages of control and elimination programmes. A research and development agenda for helminthiasis modelling is proposed based on identified gaps that need to be addressed for models to become useful decision tools that can support research and control operations effectively.

In the sixth review, “Basic Research and Enabling Technologies to Support Control and Elimination of Helminthiases”, Sara Lustigman and co-authors [85] discuss that although there has been substantial scientific advancement in our understanding of the basic biology of helminthiases, major research gaps still remain that need to be addressed to improve and update fundamental knowledge of helminth biology, and to translate such knowledge into novel intervention tools, namely, parasite genomics and functional genomics, proteomics and metabolomics, helminth immunology and (immuno)pathology, host–parasite–vector interactions, and transmission biology.

In the seventh and final review, “Health Research and Capacity Building in Disease-Endemic Countries for Helminthiases Control”, Mike Y. Osei-Atweneboana and co-authors [86] discuss the challenges confronting the building and maintenance of research capacity in disease-endemic countries, the global, regional, and national efforts and strategies towards establishing such research capacity, and the implications of regional and national health research policies on the control of helminthiases. The authors conclude that strategies for building research capacity and underlying policies are less than satisfactory in disease-endemic countries, where North-South cooperation is typically stronger than South-South collaboration. The various attempts to remedy this situation through the consolidation of links between disease-endemic regions, particularly in Africa, are discussed.

## Conclusion

The major helminth control and elimination programmes past and present have benefited from a substantial body of fundamental and operations research that has contributed to the progress and success of these programmes in the last three decades. In spite of these advancements, research investment and development for helminth infections has lagged behind the attention and resources devoted to other infectious diseases. As a result, major deficiencies exist in intervention and diagnostic tools that are appropriate to the changing demands of the large-scale preventative chemotherapy strategies that have become synonymous with helminth control, in understanding the social epidemiology and environmental ecology of these infections, in capitalising on the potential that mathematical models have to offer as decision-support instruments, and importantly, in the understanding of fundamental helminth biology that can open up avenues for novel targets and for assessing the evolutionary implications of ongoing interventions. The DRG4, through its TDR mandate, and in consultation with other disease-specific and thematic groups, and stakeholders, has identified, ranked, and projected in different time horizons, ten top priority research areas (Table S3) considered to be essential for the attainment of control and elimination efforts against helminth infections of humans. These priority areas should be addressed in parallel, as they are interconnected to one another and each one would benefit from accomplishing the remainder.

## Supporting Information

Table S1 TDR Disease-Specific and Thematic Reference Groups (DRGs/TRGs) and Their Host Countries.
(PDF)

Table S2 Original DRG4 Group Members and Subjects for White Papers and Oral Presentations for First Meeting, Burkina Faso, January 2010.
(PDF)

Table S3 Top Ten Research Priority Areas for Human Helminthiases and Ranking.
(PDF)

Table S4 Projected Basic and Operations Research Landmarks Based on the Potential Impact That Implementing the Top Ten Priority Research Areas Identified by DRG4 Would Have on Human Helminthiases, Global Health, and the Achievement of the MDGs in the Short- (1–5 y), Mid- (5–15 y), and Long- (15–25 y) Terms.
(PDF)

Text S1 Disease Reference Group in Helminthiases. Description of the meetings, stakeholder consultations, and methodology for identification, prioritisation, and ranking of research gaps.
(PDF)

Text S2 Disease Reference Group in Helminthiases. Projected time horizons for the achievement of priority research areas and potential impact on global health.
(PDF)
