# Supplementary material for: A Research Agenda for Helminth Diseases of Humans: Towards Control and Elimination
Source: PLoS Negl Trop Dis. 2012 Apr 24;6(4):e1547. doi: 10.1371/journal.pntd.0001547 (PMC3335858; doi:10.1371/journal.pntd.0001547)
Supplement: Table S1 — TDR Disease-Specific and Thematic Reference Groups (DRGs/TRGs) and Their Host Countries. (PDF) [file pntd.0001547.s001.pdf]

Boatin BA, Basáñez M-G, Prichard RK, Awadzi K, Barakat RM, García HH, Gazzinelli A, Grant WN, McCarthy J, N'Goran EK, Osei-Atweneboana MY, Sripa B, Yang G-J, Lustigman S

A Research Agenda for Helminth Diseases of Humans: Towards Control and Elimination

Table S1. TDR Disease-Specific and Thematic Reference Groups (DRGs/ TRGs) and their Host Countries

Reference group		Host institution and country
**DRG1**	Malaria	WHO Regional Office for Africa, Congo
**DRG2**	Tuberculosis, leprosy and Buruli ulcer	WHO country office, Philippines
**DRG3**	Chagas disease, human African trypanosomiasis and leishmaniasis	WHO country offices, Sudan and Brazil
**DRG4**	Helminth infections	African Programme for Onchocerciasis Control (APOC), Burkina Faso
**DRG5**	Dengue and other emerging viral diseases of public health importance	WHO country office, Cuba
**DRG6**	Zoonoses and marginalized infectious diseases of poverty	WHO Regional Office for the Eastern Mediterranean, Egypt
**TRG1**	Social sciences and gender	WHO country office, Ghana
**TRG2**	Innovation and technology platforms for health interventions in infectious diseases of poverty	WHO country office, Thailand
**TRG3**	Health systems and infectious disease control programmes	WHO country office, Nigeria
**TRG4**	Environment, agriculture and infectious diseases of poverty	WHO country office, China
