# Supplementary material for: A Research Agenda for Helminth Diseases of Humans: Towards Control and Elimination
Source: PLoS Negl Trop Dis. 2012 Apr 24;6(4):e1547. doi: 10.1371/journal.pntd.0001547 (PMC3335858; doi:10.1371/journal.pntd.0001547)
Supplement: Table S2 — Original DRG4 Group Members and Subjects for White Papers and Oral Presentations for First Meeting, Burkina Faso, January 2010. (PDF) [file pntd.0001547.s002.pdf]

Boatin BA, Basáñez M-G, Prichard RK, Awadzi K, Barakat RM, García HH, Gazzinelli A, Grant WN, McCarthy J, N'Goran EK, Osei-Atweneboana MY, Sripa B, Yang G-J, Lustigman S

A Research Agenda for Helminth Diseases of Humans: Towards Control and Elimination

Table S2. Original DRG4 Group Members and Subjects for White Papers and Oral Presentations[§] for First Meeting, Burkina Faso, January 2010

Name	Subjects for White Papers and Oral Presentations
Sara Lustigman (Chair)	Vaccines as an integrated and complementary control measure to MDA: filariases, schistosomiasis, STHs
Boakye A. Boatin (Co-Chair)	Operations research; challenges and needs to help fill programmatic gaps: onchocerciasis, LF, schistosomiasis; community-directed interventions
Kwablah Awadzi	Surveillance for drug resistance; identification of sub-optimal responders to anthelmintic treatment; clinical trials for new anti-filarial drugs
Rashida M. Barakat	Operational studies/challenges to control schistosomiasis in Africa; disease burden; diagnosis; praziquantel resistance
María-Gloria Basáñez	Epidemiological, parasitological, and transmission patterns of helminth infections; mathematical models to support control strategies and help predict the success of control interventions
Héctor H. García	Present research on taeniasis/cysticercosis; what is needed to control/prevent these infections, regional differences between continents
Andrea Gazzinelli	Control/prevention strategies of poly-parasitism; integrated control programmes; social ecology of helminthiases; gender issues; health systems
Warwick N. Grant	New tools for drug discovery and development; tools for monitoring of drug resistance
James McCarthy	Diagnosis of helminth infections; present tools for estimating disease burden; prediction of success of MDA; what is needed to support precise diagnosis when infection or disease burdens are low; integration of new tools, improvement of existing tools
Eliézer K. N'Goran	Role of geospatial tools for disease risk mapping; surveillance and predictions for resource allocation in order to achieve the goals of integrated and sustainable control measures of helminth infections in Africa; prospects and challenges of integrated control measures
Roger K. Prichard	MDA-based control measures and potential emergence of drug resistance; gaps in surveillance, parasite genetic assays in onchocerciasis, LF, STHs
Banchob Sripa	Operational studies/challenges to control/prevent trematode and cestode infections/diseases with particular emphasis on those unique to Southeast Asia vs. Africa or other regions
Guo-Jing Yang	New integrated strategies to control transmission of schistosomiasis; lessons from China and Southeast Asia; the influence of climate and environmental change on disease burden and control measures

[§]In addition to the presentations by DRG4 group members, Dr Piero L. Olliaro, in representation of the NTD Department of the WHO and TDR delivered presentations on: “Preventive chemotherapy and transmission control: issues related to research in the field of helminthiasis”; “Addressing resistance development in tropical disease pathogens”, and “Research priorities for human anthelmintics”.
