# Supplementary material for: A Research Agenda for Helminth Diseases of Humans: Towards Control and Elimination
Source: PLoS Negl Trop Dis. 2012 Apr 24;6(4):e1547. doi: 10.1371/journal.pntd.0001547 (PMC3335858; doi:10.1371/journal.pntd.0001547)
Supplement: Table S3 — Top Ten Research Priority Areas for Human Helminthiases and Ranking. (PDF) [file pntd.0001547.s003.pdf]

Boatin BA, Basáñez M-G, Prichard RK, Awadzi K, Barakat RM, García HH, Gazzinelli A, Grant WN, McCarthy J, N'Goran EK, Osei-Atweneboana MY, Sripa B, Yang G-J, Lustigman S.
A Research Agenda for Helminth Diseases of Humans: Towards Control and Elimination

Table S3. Top 10 Research Priority Areas for Human Helminthiases and Ranking

Core Theme[a]	Priority[b]	Description of Priority	Ranking[c] (1–5)	Helminthiasis-based Ranking[d]	
(1)	1	Optimize existing intervention tools to maximize impact (taking into account polyparasitism) and sustainability. The tools include pharmaceuticals, vaccines, vector control and eco-health approaches (access to clean water and sanitation, improved nutrition, education). Sustainability depends on minimizing selection for drug resistance and maintaining community support for adequate coverage and compliance	4.3	Onchocerciasis	4.9
				LF	4.4
				STHs	4.7
				Schistosomiasis	4.5
				Food-borne trematodiases	3.6
				Taeniasis/ cysticercosis	3.6
	2	Develop novel control tools which will improve impact and sustainability. The tools include new pharmaceuticals, vaccines, vector control methods and eco-health approaches	4.1	Onchocerciasis	4.5
				LF	4.2
				STHs	4.3
				Schistosomiasis	4.3
				Food-borne trematodiasis	3.7
				Taeniasis/ cysticercosis	3.7
(2)	3	Improve existing/develop novel diagnostic tests, with particular reference to their performance regarding sensitivity, specificity, multiplex capacity and ability to measure infection intensity/active infection. Sensitivity and specificity are mostly important to enable diagnosis of infection at low prevalence in elimination settings and to confirm cure/absence of particular infection	4.1	Onchocerciasis	4.9
				LF	4.2
				STHs	4.6
				Schistosomiasis	4.5
				Food-borne trematodiases	3.6
				Taeniasis/ cysticercosis	3.3
	4	Standardize and validate methodologies and protocols for diagnosis in monitoring and evaluation (M&E) settings	3.9	Onchocerciasis	4.6
				LF	4.3
				STHs	4.5
				Schistosomiasis	4.3
				Food-borne trematodiases	2.7
				Taeniasis/ cysticercosis	3.3
(1, 3)	5	Develop strategies incorporating delivery of multiple and combinations of interventions at various (individual, community, district, national) levels to maximize sustainability of control programmes in general and of integrated control programmes in particular	4.6	Onchocerciasis	4.9
				LF	4.9
				STHs	4.8
				Schistosomiasis	5.0
				Food-borne trematodiases	3.8
				Taeniasis/ cysticercosis	4.1
(3)	6	Develop strategies (taking gender issues into account) to increase community participation, ownership and empowerment, as well as equity in access by communities and risk groups to health services	4.7	Onchocerciasis	4.7
				LF	4.7
				STHs	4.8
				Schistosomiasis	4.9
				Food-borne trematodiases	4.2
				Taeniasis/ cysticercosis	4.7

(4)	7	Develop and refine mathematical models to investigate relationships between infection and morbidities to aid programmes aiming to reduce the burden of disease (elimination of public health problem). Such models need to take into account cumulative effects of chronic disease for evaluation of disease burden and the impact on such burden of control interventions	3.6	Onchocerciasis	4.0
				LF	3.9
				STHs	3.7
				Schistosomiasis	4.0
				Food-borne trematodiases	2.8
				Taeniasis/ cysticercosis	3.1
	8	Increase use and application of mathematical models to aid M&E, surveillance, elimination efforts, and the design of sampling protocols as well as the monitoring of intervention efficacy including drug resistance. These models should be linked to economic impact studies of the diseases and cost-effectiveness analyses of the interventions, their combinations and their alternatives	4.0	Onchocerciasis	4.3
				LF	4.3
				STHs	4.3
				Schistosomiasis	4.1
				Food-borne trematodiases	3.4
				Taeniasis/ cysticercosis	3.4
(5)	9	Define the determinants and impact of parasite modulation of the host-parasite relationship, including impact on the host response to concurrent infection with other helminth and non-helminth pathogens and to vaccination, and parasite responses, including immune responses to interventions	3.9	Onchocerciasis	4.3
				LF	4.4
				STHs	4.1
				Schistosomiasis	4.3
				Food-borne trematodiases	3.2
				Taeniasis/ cysticercosis	3.5
	10	Annotate parasite genomes and transcriptomes and develop tools for parasite functional genomics (and other 'omics') in key species	4.1	Onchocerciasis	4.8
				LF	4.4
				STHs	4.2
				Schistosomiasis	3.9
				Food-borne trematodiases	3.5
				Taeniasis/ cysticercosis	3.7

[a] Core themes are: (1) Control Interventions; (2) Epidemiology and Surveillance; (3) Environmental and Social Ecology; (4) Data and Modelling; (5) Basic (Fundamental) Biology (see Figure 1 in main text).

[b] Numbering of the top 10 priories does not reflect order of importance; instead they are organized according to core theme; all the (inter-connected) priorities are to be addressed in parallel as each priority will benefit from accomplishing the others.

[c] The arithmetic mean, across the 6 helminthiases under consideration, of the scores (from 1 to 5) given by each member of the DRG4 group independently.

[d] The arithmetic mean, for each of the helminthiases, of the scores (1–5) given by each member of the DRG4 group independently.

1: No priority; 2: Low priority; 3: Moderate priority; 4: High priority; 5: Essential.
