# Supplementary material for: A Research Agenda for Helminth Diseases of Humans: Towards Control and Elimination
Source: PLoS Negl Trop Dis. 2012 Apr 24;6(4):e1547. doi: 10.1371/journal.pntd.0001547 (PMC3335858; doi:10.1371/journal.pntd.0001547)
Supplement: Table S4 — Projected Basic and Operations Research Landmarks Based on the Potential Impact That Implementing the Top Ten Priority Research Areas Identified by DRG4 Would Have on Human Helminthiases, Global Health, and the Achievement of the MDGs in the Short- (1–5 y), Mid- (5–15 y), and Long- (15–25 y) Terms. (PDF) [file pntd.0001547.s004.pdf]

Table S4. Projected Basic and Operations Research Landmarks based on the Potential Impact that Implementing the Top 10 Priority Research Areas Identified by DRG4 would have on Human Helminthiases, Global Health, and the Achievement of the MDGs‡ in the Short- (1– 5 yr), Mid- (5–15 yr) and Long- (15–25 yr) Terms

Potential impacts of the top 10 research priorities identified by DRG4 in the following time-horizons									
	5 years			15 years			25 years		
	Local	National	Regional	Local	National	Regional	Local	National	Regional
**Overall, including control of infectious diseases of poverty**<br><br>**(MDGs1–8)**	Improved health literacy<br><br>Increased capacity building and interdisciplinary research			Much improved health literacy and education, integrated health intervention for polyparasitism, multi- and interdisciplinary research<br><br>Elimination of onchocerciasis & LF as public health problems in many foci and elimination of infection in some foci			Equity in health literacy, improved universal access to interventions<br><br>Fully integrated health interventions<br><br>Major reduction of disease burden due to helminthiases; elimination of infection where feasible		
**Technological innovations**<br><br>**(MDGs6,8)**	Mobile phone- and PDA-based reporting systems (mapping, GIS, and surveillance)	Optimized (existing) drug combinations and treatment regimens<br><br>Refined models for infection and morbidity relationships<br><br>Improved diagnostic tests (sensitivity, specificity) for low intensity infections<br><br>(Bio)markers for drug resistance		Implemented use of novel diagnostics for low intensity/ active infections<br><br>Improved operational MDA strategies that use markers of drug resistance	Tools for parasite functional genomics in key species<br><br>Accurate burden of disease/ infection are defined	Novel drugs, vaccines, and vector control tools from bio-technology<br><br>Partnerships produce drugs, vaccines and (bio)marker assays	Disease-endemic countries produce own drugs, vaccines, (bio)markers and vector control tools<br><br>Equity in health technology in disease-endemic countries		
**Health systems and decision support to programmes**<br><br>**(MDGs3,6,8)**	Increased community participation, ownership and empowerment<br><br>New improved community-directed interventions	Standardized and validated methodologies and protocols for diagnosis in M&E settings<br><br>Epidemiological models to aid M&E, surveillance, and elimination efforts are developed<br><br>Impact of intervention on the host immune response can be taken into consideration for MDA operational strategies		Maximized commitment to sustainability of control programmes in general and to integrated NTD control in particular<br><br>Models for co-infection and for the impact of climate change on helminth infections are developed to support health systems decisions<br><br>Models to support M&E, surveillance and elimination efforts continue to be refined and used by control programmes			Improved equity in access by communities and risk groups to health services including gender issues	Integrated NTD control fully in place as well as integration with other interventions for infectious disease	

Table S4. Continued

Potential impacts of the top 10 research priorities identified by DRG4 in the following time-horizons									
	**5 years**			**15 years**			**25 years**		
	**Local**	**National**	**Regional**	**Local**	**National**	**Regional**	**Local**	**National**	**Regional**
**Environment**<br><br>**(MDGs1,3–7)**	Implementation of water and sanitation strategies (community-led total sanitation)	Models for the impact of climate change on helminth infections are developed	Vaccines against zoonotic reservoirs (e.g. pigs, bovines)<br><br>Better and more complex early warning systems, incl. disaster preparedness for more extreme weather				Sanitation for all in disease-endemic countries, markedly improved water management		

[‡]**Millennium Development Goals. MDG1:** Eradicate extreme poverty and hunger; **MDG2:** Achieve universal primary education; **MDG3:** Promote gender equality and empower women; **MDG4:** Reduce child mortality; **MDG5:** Improve maternal health; **MDG6:** Combat HIV/AIDS, malaria and other diseases; **MDG7:** Ensure environmental sustainability; **MDG8:** Develop a global partnership for development.
