# Supplementary material for: A Research Agenda for Helminth Diseases of Humans: Towards Control and Elimination
Source: PLoS Negl Trop Dis. 2012 Apr 24;6(4):e1547. doi: 10.1371/journal.pntd.0001547 (PMC3335858; doi:10.1371/journal.pntd.0001547)
Supplement: Text S1 — Disease Reference Group in Helminthiases. Description of the meetings, stakeholder consultations, and methodology for identification, prioritisation, and ranking of research gaps. (PDF) [file pntd.0001547.s005.pdf]

Boatin BA, Basáñez M-G, Prichard RK, Awadzi K, Barakat RM, García HH, Gazzinelli A, Grant WN, McCarthy J, N'Goran EK, Osei-Atweneboana MY, Sripa B, Yang G-J, Lustigman S

A Research Agenda for Helminth Diseases of Humans: Towards Control and Elimination

Text S1. Disease Reference Group in Helminthiases. Description of the Meetings, Stakeholder Consultations and Methodology for Identification, Prioritization and Ranking of Research Gaps

The Disease Reference Group on Helminthiases first met from the 11th to the 16th of January, 2010 in Ouagadougou, Burkina Faso, at the headquarters of the African Programme for Onchocerciasis Control (APOC) (Table S1). The sessions were preceded by a local stakeholder consultation meeting, after which the DRG4 members discussed subject-specific white papers that had been commissioned by the TDR (Table S2) and prepared by the group members on research subjects ranging from basic helminth biology, epidemiology, diagnosis, intervention tools, mathematical modelling, social and behavioural issues pertinent to control and intervention programmes, to issues related to environmental and climate change. This discussion was followed by gap analysis and identification of major research priorities.

Periodic regional and national stakeholder consultations were part of the analysis process, enabling validation, endorsement and uptake of final research priorities, thus ensuring that the work of the group is authoritative, scientifically credible, and policy relevant. To ensure that the countries most affected by diseases of poverty contribute to, and share ownership of, the research agenda emerging from this initiative, the reference group was hosted by disease-endemic countries, in partnership with WHO country and regional offices. The first stakeholder consultation in Burkina Faso included the Ministry of Health, Helen Keller International, RISEAL (Réseau International Schistosomiases Environnement Aménagements et Lutte) /Burkina Faso (http://www.riseal.org/), United Nations Children Fund Office (UNICEF), WHO/Multi Disease Surveillance Center, and APOC, the host organization.

Based on the five core themes identified at the end of the Burkina Faso meeting listed in the main text and depicted in Figure 1, namely: 1) Interventions, 2) Epidemiology and Surveillance, 3) Environmental and Social Ecology, 4) Data and Modelling, and 5) Basic (Fundamental) Biology, five working sub-groups were organized to prepare more detailed reports indicating their first set of priority research issues as well as recommendations on each of the themes. Each group was asked to provide for each core theme: a) an overview of factors driving the persistence and re-emergence of helminth infections of humans; b) an overview of significant recent scientific advances in the field; c) a deeper analysis of control challenges/research issues by particular disease/risk factors; and d) an analysis of the research gaps accompanied by identification of priorities [1,2].

The reports from the sub-groups were reviewed by all the members of the DRG4 to arrive at a consensus on the identified priority research issues and recommendations pertaining to each of the core themes. Some members of the sub-groups met on several occasions for discussions and report writing (e.g. BAB, M-GB, RKP and SL from 5th to 9th of April, 2010 in New York) while there was continuous dialogue between all members, through teleconferences, and other means of electronic communication.

A second stakeholder consultation meeting, which preceded the second formal meeting of the group in Rio de Janeiro, Brazil, between the 8th to the 11th of October 2010, discussed the draft composite report and provided feedback on the research priorities identified. Following this, the final research gaps in each of the five core themes were identified and ranked by the DRG4 members. At the second stakeholder consultation in Rio de Janeiro, participants included the Ministry of Health of Brazil, WHO/Pan American Health Organization (PAHO), researchers from the Oswaldo Cruz Institute, and other international and local experts attending the International Schistosomiasis Symposium in Rio de Janeiro.

Other consulted stakeholders were from regional committees in Latin America and South East Asia [3–5]. Finally, in November 2010, some DRG4 members (M-GB, RKP,

JMC) met at Imperial College London to discuss the structure of the reviews presented in this series.

Identification, Prioritization, and Ranking of Research Gaps

At all stages, the DRG4 was guided by the context, comprehensiveness of approach, inclusiveness, extensive information gathering, criteria and methods for deciding on priorities discussed in [2] (both including consensus– and Delphi–based metrics). The first full draft report from the DRG4, contained 90 unranked research areas and was reviewed in detail by each member of the group, as well as by the chairs and co-chairs of the other DRGs and TRGs (Table S1), and by the stakeholders.

At the second full meeting of the DRG4 in Brazil (BAB, M-GB, RKP, WNG, BS, RB, HHG, MYO-A, AG, JMC, SL), and together with PAHO representatives and under TDR stewardship, the final ranking of the priority research areas was agreed and feasible time horizons for the achievement of each research activity/potential impact on global health were projected (described in Supplementary Text S2).

Each DRG4 member ranked independently the priority research areas identified within the five core themes and between the six helminthiases. They scored the priorities from 1 to 5, with 5 corresponding to the highest (essential) priority. The arithmetic means of the scores from each member of the group for the research areas in each core theme and for each of the helminth diseases considered were taken. The two research areas with the highest mean scores within each of the five core themes were then selected to provide 10 top priority research areas (Table 1 of the main text). A league table was prepared for the 10 priority research areas using the individual scores as criteria (Table S3).

References

1. National Institutes of Health (2007) Neglected Tropical Diseases: Identifying research gaps and opportunities. Report of a workshop held on September 27, 2007. National Institute of Allergy and Infectious Diseases, Division of Microbiology and Infectious Diseases

http://www.niaid.nih.gov/topics/tropicalDiseases/Documents/ntd.pdf

2. Viergever RF, Olifson S, Ghaffar A, Terry RF (2010) A checklist for health research priority setting: nine common themes of good practice. *Health Res Policy Syst* 8: 36.

3. Colley DG, WE Secor (2007) A schistosomiasis research agenda. *PLoS Negl Trop Dis* 1: e32.

4. Dujardin JC, Herrera S, do Rosario V, Arevalo J, Boelaert M, et al. (2010) Research priorities for neglected infectious diseases in Latin America and the Caribbean region. *PLoS Negl Trop Dis* 4: e780.

5. World Health Organization (2011) Report of the WHO Expert Consultation on foodborne trematode infections and taeniasis/cysticercosis. Vientiane, Lao People's Democratic Republic 12-16 October 2009. http://www.who.int/neglected_diseases/preventive_chemotherapy/WHO_HTM_NTD_PCT_2011.3.pdf
