# Supplementary material for: A Research Agenda for Helminth Diseases of Humans: Towards Control and Elimination
Source: PLoS Negl Trop Dis. 2012 Apr 24;6(4):e1547. doi: 10.1371/journal.pntd.0001547 (PMC3335858; doi:10.1371/journal.pntd.0001547)
Supplement: Text S2 — Disease Reference Group in Helminthiases. Projected time horizons for the achievement of priority research areas and potential impact on global health. (PDF) [file pntd.0001547.s006.pdf]

Boatin BA, Basáñez M-G, Prichard RK, Awadzi K, Barakat RM, García HH, Gazzinelli A, Grant WN, McCarthy J, N'Goran EK, Osei-Atweneboana MY, Sripa B, Yang G-J, Lustigman S

A Research Agenda for Helminth Diseases of Humans: Towards Control and Elimination

Text S2. Disease Reference Group in Helminthiases. Projected Time-Horizons for the Achievement of Priority Research Areas and Potential Impact on Global Health

Each member of the DRG4 group was asked to set hypothetical time-frames for the projected achievement of each of the top 10 priority areas identified (Table 1 of the main text) in time horizons of 5, 10, 15, 20 and 25 year periods. These time-lines were then collated into timeframe periods of 1–5 years (short-term), 5–15 years (medium-term), and 15–25 years (long-term). These time-frames were then used to project the impact that the attainment of these research landmarks would have on the control and elimination of human helminth infections in particular, and more generally on the improvement of global health and the attainment of the Millennium Development Goals[1], taking into consideration their impact on disease burden, technological innovation, health systems, decision support to helminth control programmes, and the environment. The outcomes are presented in Table S4. Control of infectious diseases of poverty (MDG1) should effect an appreciable improvement on health literacy, community empowerment, and research capacity in disease-endemic countries in the short- through to the long-term (MDGs2,3,8), whereas research on burden of morbidity and mortality, and the impact of this research on populations (MDGs4,5) would require a longer-term horizon. It is anticipated that optimized combinations of existing dugs will be available and having an appreciable impact in the next 5 years (MDG6), whilst the full impact of integrated control with that of other NTDs and infectious diseases (MDGs1,4–6,8) will only be realized in the long-term. In the area of technological innovation, the development of novel drugs and combinations for individual, targeted and mass treatment

[1] MDG1: Eradicate extreme poverty and hunger; MDG2: Achieve universal primary education; MDG3: Promote gender equality and empower women; MDG4: Reduce child mortality; MDG5: Improve maternal health; MDG6: Combat HIV/AIDS, malaria and other diseases; MDG7: Ensure environmental sustainability; MDG8: Develop a global partnership for development.

(MDGs4–6) is foreseen in the long-term (due to the prolonged horizons from drug discovery to safe use in populations). Likewise, whist diagnostic improvement may be achieved with existing assays in the short-term, the development and impact of (bio)markers (e.g. from metabolomics) for detection of active infection (MDG6) may be accomplished in the medium- and long-terms. It is envisaged that mathematical models to aid the design and analysis of M&E protocols can be developed/applied in the short-term, with those for supporting surveillance and elimination efforts (MDG6,8) being used and refined in the medium-term. Models for the relationship between infection and morbidity are seen as a (feasible) priority in the short-term to support global burden of disease assessment efforts, but their refinement, and use for the investigation and evaluation of co-morbidities due to polyparasitism (MDGs4,5) will extend into the medium- and long-terms.
